# When an Intrauterine Pregnancy Is Not Reassuring: Spontaneous Heterotopic Pregnancy With Tubal Rupture

**DOI:** 10.14740/jmc5385

**Published:** 2026-09-04

**Authors:** Nikoleta Stoyanova, Rebecca Caiulo, Nikola Popovski

**Affiliations:** a Department of Obstetrics and Gynecology, Medical University Pleven, Pleven, Bulgaria; b Clinic of Obstetrics and Gynecology, UMHAT “Dr. Georgi Stranski”, Pleven, Bulgaria

**Keywords:** Heterotopic pregnancy, Spontaneous conception, Ectopic pregnancy, Tubal rupture, Hemoperitoneum

## Abstract

Heterotopic pregnancy (HP), defined as the co-existence of intrauterine and ectopic gestations, is a rare but potentially life-threatening condition. While its incidence has increased with the widespread use of assisted reproductive technologies (ARTs), spontaneous HP remains uncommon and may be overlooked when an intrauterine pregnancy (IUP) is identified. We report the case of a 22-year-old multi-parous woman who presented at 5 weeks and 3 days of amenorrhea with acute pelvic pain, mild vaginal bleeding, hypotension, and signs of an acute abdomen. Transvaginal ultrasonography (TVUS) demonstrated a non-viable IUP, a right adnexal gestational sac containing a yolk sac, and free fluid in the pouch of Douglas. Emergency laparotomy revealed a ruptured right tubal ectopic pregnancy (EP) with hemoperitoneum. Right salpingectomy and uterine curettage were performed, and histopathological examination confirmed HP. The post-operative course was uneventful. A review of recently published cases indicates that abdominal pain and vaginal bleeding are the most common presenting symptoms, with tubal implantation representing the predominant ectopic location. Clinicians should maintain a high index of suspicion for HP in symptomatic early pregnancies, regardless of risk factors or confirmation of an intrauterine gestation. Prompt diagnosis and timely surgical management are essential to reduce maternal morbidity and prevent life-threatening complications.

## Introduction

Heterotopic pregnancy (HP), also referred to as combined pregnancy, is defined as the co-existence of an intrauterine pregnancy (IUP) and an ectopic pregnancy (EP) [1]. While it can occur following spontaneous conception, its incidence is significantly higher in association with assisted reproductive technologies (ARTs), particularly *in vitro* fertilization (IVF). The reported incidence of HP ranges from approximately one in 8,000 to one in 30,000 pregnancies in natural conception cycles, with substantially higher rates observed in ART populations [2].

HP represents a significant diagnostic and therapeutic challenge for practitioners, as the presence of an IUP may lead clinicians to overlook a concurrent EP. Delayed or missed diagnosis can result in life-threatening complications, including rupture of the EP, intra-abdominal hemorrhage, and hypovolemic shock.

Known risk factors for HP include the use of ART, particularly in cases with tubal factor infertility, a history of previous EP, multiple prior abortions, tubal damage associated with pelvic inflammatory disease (PID) and prior pelvic or tubal surgery [3, 4]. However, HP may also occur in women without identifiable risk factors, suggesting that idiopathic cases are possible.

The clinical presentation of HP is often variable, ranging from non-specific symptoms such as mild abdominal pain or vaginal bleeding, to acute presentations including adnexal tenderness and signs of intraperitoneal hemorrhage. In some cases, patients may present with life-threatening complications such as hypovolemic shock secondary to tubal rupture and intra-abdominal bleeding; however, some cases remain asymptomatic, particularly in early stages.

Early diagnosis is crucial to prevent serious complications, also aiming to preserve the IUP whenever possible. The diagnosis of HP remains challenging, as the confirmation of an IUP may lead to a false sense of re-assurance and delay the identification of a concurrent EP. Transvaginal ultrasound (TVUS) is the primary diagnostic tool, allowing for an evaluation of the intrauterine cavity and both adnexa. Magnetic resonance imaging (MRI) may serve as a useful adjunctive diagnostic modality in hemodynamically stable patients when ultrasonographic findings are inconclusive or when additional characterization of abdominal pathology is necessary. Nevertheless, in the presence of clinical deterioration or signs suggestive of an acute abdomen, surgical intervention should not be delayed, as early operative management is essential to prevent severe hemorrhage and associated maternal complications.

We present a case of spontaneous HP complicated by a ruptured tubal EP and a non-viable intrauterine gestation. To place our findings in context, we also performed a brief literature review of full-text case reports on spontaneously conceived HP published during the last 5 years and identified through PubMed.

## Case Report

### Investigations

A 22-year-old woman, gravida 4 para 3 (G4P3), was admitted to the Department of Gynecology with acute pelvic pain and mild vaginal bleeding at 5 weeks and 3 days of amenorrhea. Her obstetrical history included one vaginal delivery in 2021, one cesarean section performed in 2022 due to breech presentation and fetal distress, and one successful vaginal birth after cesarean section in 2024. She denied previous pelvic surgery, chronic medical conditions, or current medication use. The patient also denied ovulation induction or use of assisted ARTs. The family history was unremarkable.

### Diagnosis

On admission, the patient presented with hypotension (90/70 mm Hg), tachycardia (110 beats/min), and clinical signs of acute abdomen. Gynecological examination revealed a mildly enlarged and tender uterus, mild uterine bleeding, and marked tenderness in the pouch of Douglas.

**Figure 1:**
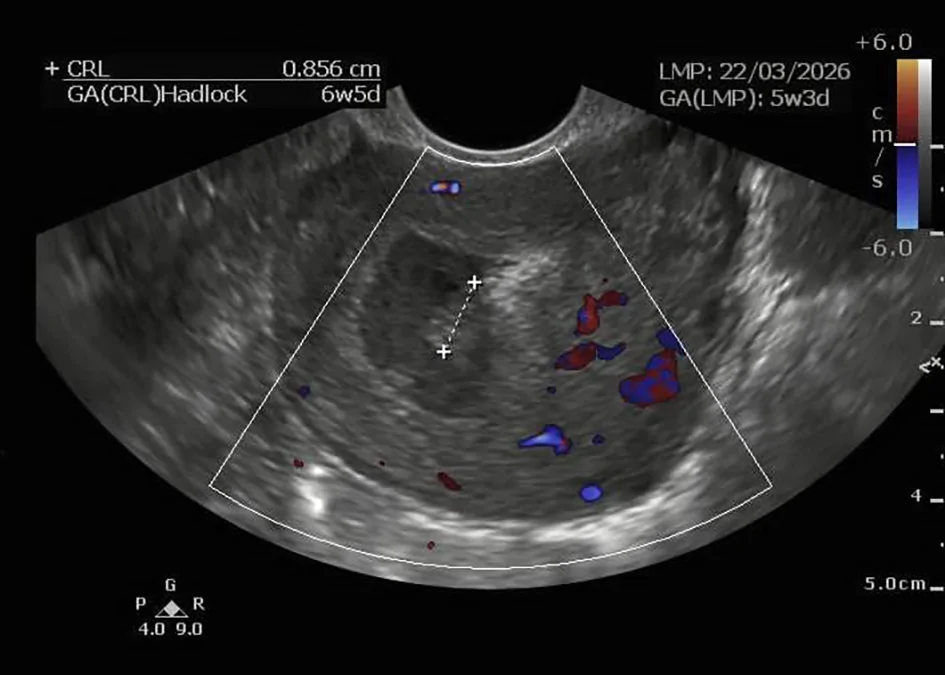
Transvaginal ultrasound demonstrating an intrauterine embryo with a CRL of 8.5 mm and absent cardiac activity, consistent with missed abortion. CRL: crown-rump length.

**Figure 2:**
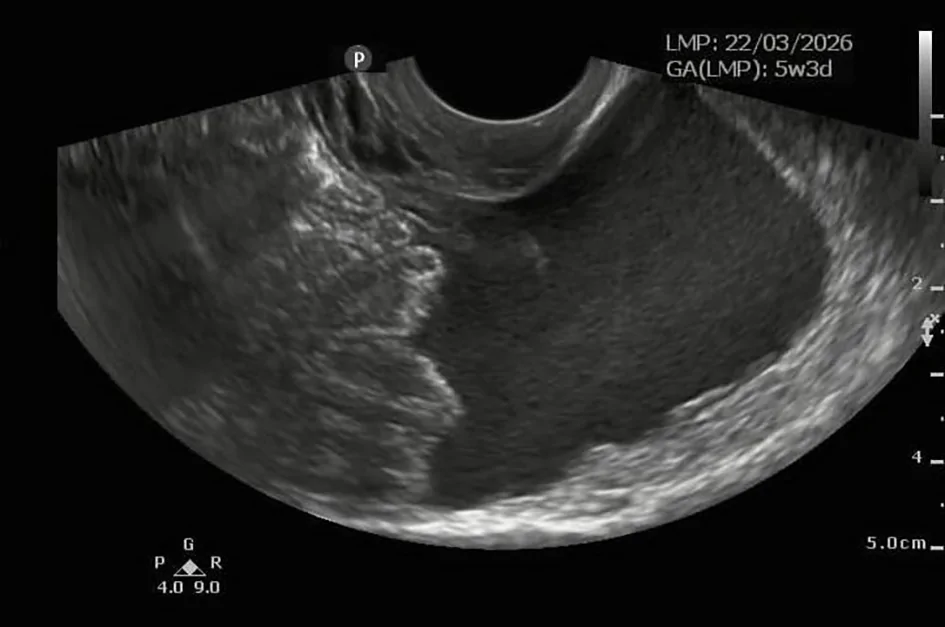
Free fluid within the pouch of Douglas identified on transvaginal ultrasound, suggestive of hemoperitoneum.

**Figure 3:**
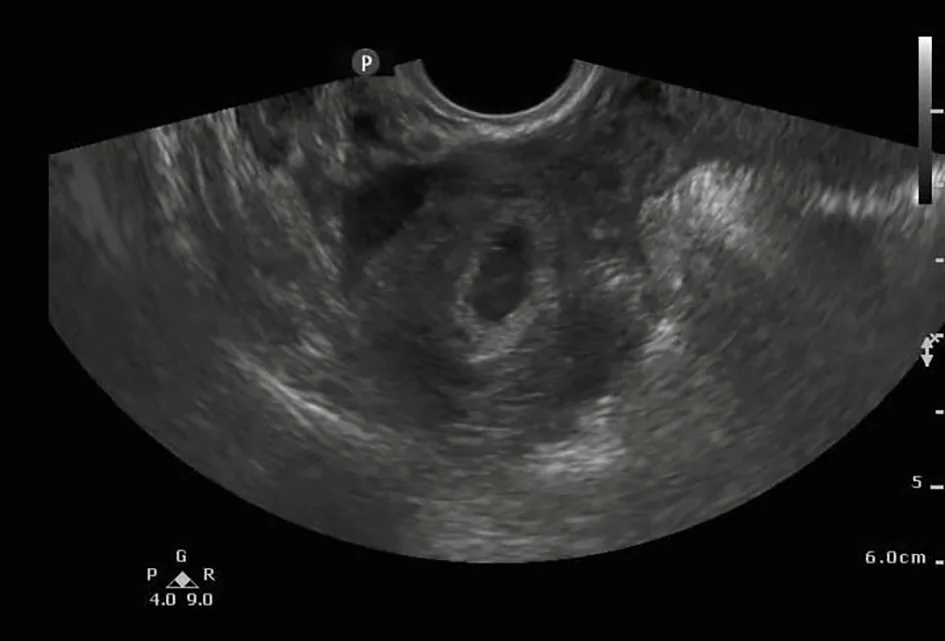
Right adnexal ectopic gestational sac containing a yolk sac, consistent with tubal pregnancy.

TVUS demonstrated an intrauterine gestational sac containing an embryo with a crown-rump length (CRL) of 8.5 mm, corresponding to approximately 6 weeks of gestation, with no detectable cardiac activity, consistent with a non-viable IUP (Fig. 1). A moderate amount of free fluid was visualized in the pouch of Douglas (Fig. 2). Further examination of the adnexa revealed a right tubal ectopic gestational sac containing a yolk sac (Fig. 3).

Laboratory investigations demonstrated mild anemia and leukocytosis, while the remaining laboratory parameters were within normal limits.

A diagnosis of HP with ruptured tubal pregnancy and concurrent non-viable IUP was established.

### Treatment

**Figure 4:**
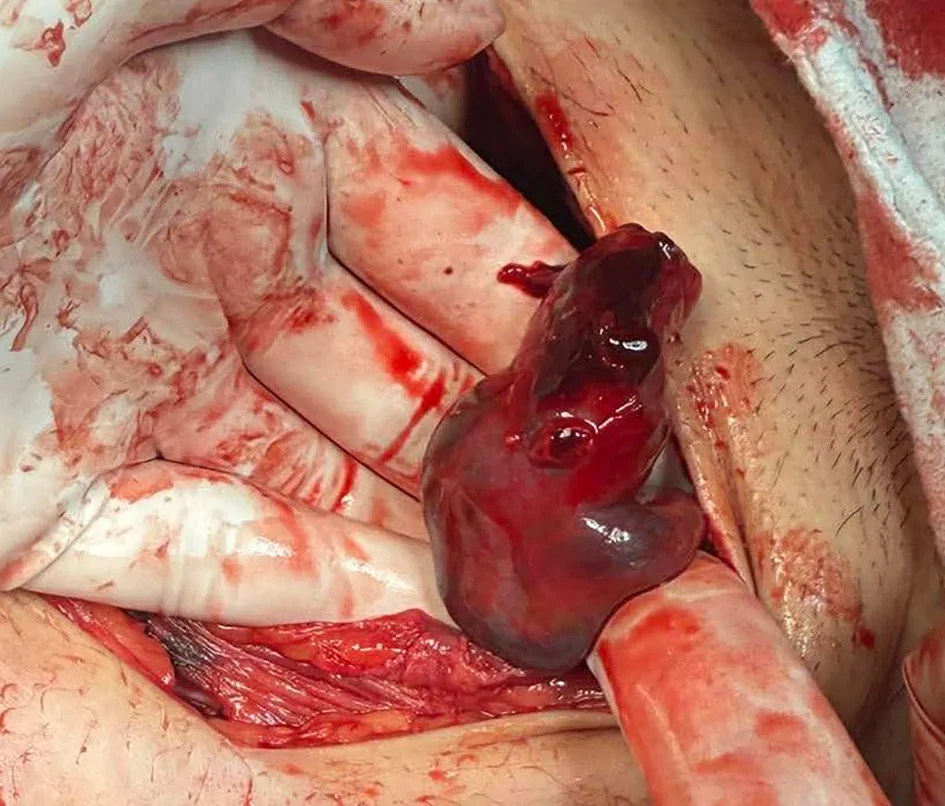
Intra-operative findings demonstrating a ruptured right tubal ectopic pregnancy.

Emergency exploratory laparotomy was performed under general anesthesia. Approximately 500 mL of blood and blood clots were found within the abdominal cavity due to a ruptured EP in the right fallopian tube (Fig. 4).

Right salpingectomy and peritoneal lavage were performed. Uterine curettage was additionally carried out to evacuate the missed abortion. Upon the patient's request and after obtaining written informed consent, sterilization of the left fallopian tube was also performed.

### Follow-up and outcomes

The post-operative course was uneventful. The patient remained hemodynamically stable and demonstrated progressive clinical improvement. Broad-spectrum antibiotic therapy was administered with ceftriaxone 2 g intravenously twice daily and metronidazole 500 mg intravenously three times daily. Intravenous fluid support consisted of 2,000 mL of balanced crystalloid solution. No post-operative complications were observed, and the patient recovered satisfactorily during hospitalization, with discharge on post-operative day 4. Histopathological examination confirmed the simultaneous presence of intrauterine and extrauterine gestations.

The key clinical, ultrasonographic, surgical, and histopathological findings of the present case are summarized in Table 1. This study was conducted in accordance with the Declaration of Helsinki. The ethical approval has been waived by the Institutional Review Board of Medical University Pleven for reporting individual cases or case series when informed consent is provided by the patient.

**Table 1:** Clinical Summary of the Present Case

Variable	Findings
Age	22 years
Gravidity/parity	G4P3
Conception	Spontaneous
Risk factors for EP	None identified
Symptoms	Lower abdominal pain, vaginal bleeding
Hemodynamic status	BP 90/70 mm Hg, HR 110 bpm
TVUS findings (IUP)	CRL 8.5 mm (6 + 5 weeks), absent cardiac activity
TVUS findings (EP)	Right adnexal gestational sac with yolk sac (6 weeks)
Associated findings	Free fluid in pouch of Douglas/hemoperitoneum
Diagnosis	Spontaneous heterotopic pregnancy with ruptured right tubal ectopic pregnancy and missed abortion
Surgical treatment	Exploratory laparotomy + right salpingectomy + uterine curettage
Histopathology	Confirmed tubal ectopic pregnancy with chorionic villi

G4P3: gravida 4 para 3; EP: ectopic pregnancy; CRL: crown-rump length; IUP: intrauterine pregnancy; HR: heart rate; BP: blood pressure; TVUS: transvaginal ultrasound.

## Discussion

The present case describes a spontaneous HP in a 22-year-old woman without identifiable risk factors, presenting with a ruptured tubal EP, hemoperitoneum, and a concurrent non-viable IUP requiring emergency laparotomy. This presentation illustrates the diagnostic challenge of spontaneous HP and highlights the potential for life-threatening complications despite the absence of traditional risk factors.

Although HP is strongly associated with ART, spontaneous cases remain extremely rare. Historically, the earliest documented case of HP is generally attributed to the French anatomist and surgeon Joseph Guichard Duverney, who described the condition in 1708 as an autopsy finding, underscoring the longstanding diagnostic limitations of this entity prior to the advent of modern imaging techniques. Since its first description, advances in TVUS and reproductive medicine have significantly improved the detection of HP. Nevertheless, spontaneous HP continues to represent an uncommon clinical entity. The present case is notable because no identifiable risk factors including ART, PID, previous EP, or tubal surgery were present.

One of the major diagnostic challenges in HP is the presence of a confirmed IUP, which may create a false sense of reassurance and lead clinicians to overlook a concurrent ectopic gestation. As a result, evaluation of the adnexa may be incomplete or insufficiently detailed, particularly in early pregnancy. Furthermore, the sensitivity of TVUS for detecting HP is lower during the early gestational period, especially when adnexal findings are subtle or atypical. In such cases, the presence of free intraperitoneal fluid, particularly in symptomatic patients presenting with abdominal pain, should raise suspicion for tubal rupture and intra-abdominal hemorrhage. Therefore, the visualization of an IUP should never exclude systematic examination of both adnexa, especially in patients presenting with acute abdominal symptoms.

To better contextualize the present case, a brief literature review of published reports describing spontaneous HP was performed, and the main clinical characteristics are summarized in Table 2 [5–35].

**Table 2:** Summary of Reviewed Case Reports of Spontaneous Heterotopic Pregnancy

Author	Age	Risk factors	GA at diagnosis	Presentation	Ectopic location	Rupture	IUP outcome	Management
Bouab M, 2024 [5]	28	None	10 weeks	Acute pelvic pain, vaginal bleeding	Tubal	Ruptured	Miscarriage	Salpingectomy
Nguyen KP, 2022 [6]	27	None	5 + 5 weeks	Acute pelvic pain, vaginal bleeding	Tubal	Ruptured	Early pregnancy loss at 8 weeks	Laparoscopic salpingectomy
Dao D, 2023 [7]	NA	None	NA	Acute pelvic pain, vomiting	Tubal	Non-ruptured	Termination by patient’s desire	Laparoscopic salpingectomy
Valencia V, 2024 [8]	37	None	7 weeks	Acute abdominal pain, vaginal bleeding	Tubal	Non-ruptured	NA	Salpingectomy
Lober R, 2024 [9]	36	None	6 weeks	Acute abdominal pain	Ovarian	Ruptured	Vaginal delivery at 38 weeks	Laparoscopic removal of the ovarian mass
Khouloud M, 2024 [10]	36	None	12 weeks	Pelvic tenderness, vaginal bleeding	Abdominal	Non-ruptured	Miscarriage at 6 weeks	Laparotomy
Maharjan S, 2023 [11]	28	None	8 + 3 weeks	Abdominal pain, vaginal spotting	Tubal	Non-ruptured	Vaginal delivery at 38 weeks	Laparoscopic salpingectomy
Momtahan M, 2021 [12]	31	PID	36 + 4 weeks	Labor pain	Abdominal	Alive fetus weighing 2,200 g	Cesarean section	Operational delivery of both fetuses
Ouafidi B, 2021 [13]	32	None	9 + 2 weeks	Acute pelvic pain	Tubal	Ruptured	Vaginal delivery at 37 + 5 weeks	Salpingectomy
Maree G, 2022 [14]	37	None	12 weeks	Lower abdominal pain, intermittent vaginal bleeding	Ovarian	Non-ruptured	Uterine curettage	Ovarian excision
Abdelmonem AH, 2021 [15]	38	None	9 + 5 weeks	Abdominal pain, nausea, vomiting	Tubal	Non-ruptured	NA	Laparoscopic salpingectomy
Wang F, 2025 [16]	36	None	12 weeks	Abdominal pain	Tubal	Ruptured	Termination by patient’s desire	Laparoscopic salpingectomy
Regragui A, 2025 [17]	41	Prior tubal pregnancy	5 + 4 weeks	Signs of hemorrhagic shock	Tubal	Ruptured	Uterine curettage due to missed abortion	Salpingectomy
Kathopoulis N, 2023 [18]	26	None	17 + 2 weeks	Lower abdominal pain	Tubal	Ruptured	Cesarean section at 38 weeks	Laparoscopic salpingectomy
Guraslan H, 2024 [19]	29	NA	7 + 6 weeks	Asymptomatic	Cesarean scar pregnancy	Non-ruptured	Cesarean section at 38 weeks	Laparoscopic excision of the scar pregnancy
Tariq Ahmed Alabsi M, 2024 [20]	34	None	5 + 5 weeks	Asymptomatic	Cesarean scar pregnancy	Non-ruptured	Cesarean section at 38 weeks	Spontaneous resolution of the scar pregnancy
Takoutsing B, 2025 [21]	31	None	11 + 6 weeks	Crampy abdominal pain, vaginal spotting	Tubal	Ruptured	NA	Salpingectomy
Nasrollahi F, 2024 [22]	28	None	8 weeks	Shortness of breath, chest and abdominal pain, syncope	Tubal	Ruptured	Term delivery	Salpingectomy
Rodriguez MS, 2026 [23]	33	None	7 + 1 weeks	Pelvic pain, vaginal bleeding	Tubal	Non-ruptured	Cesarean section at 38 weeks	Laparoscopic salpingectomy
Dubbewar A, 2022 [24]	40	None	NA	Abdominal pain	Tubal	Ruptured	Evacuation due to GTD	Laparoscopic salpingectomy
Teka H, 2023 [25]	26	None	7 + 5 weeks	Abdominal pain	Tubal	Ruptured	Viable IUP	Salpingo-oophorectomy
Ozawa N, 2021 [26]	36	None	5 + 6 weeks	Abdominal pain	Abdominal	Ruptured	Term delivery	Extraction of the abdominal mass
Kansal M, 2023 [27]	27	None	6 + 5 weeks	Pelvic pain	Tubal	Non-ruptured	Term delivery	Laparoscopic salpingectomy
Prucha Leite N, 2025 [28]	38	None	8 + 5 weeks	Acute pelvic pain, signs of hypovolemic shock	Tubal	Ruptured	Uterine curettage	Laparoscopic salpingectomy
Areys HM, 2025 [29]	22	Female genital mutilation	10 + 2 weeks	Lower abdominal pain, vomiting	Tubal	Ruptured	Term delivery	Salpingectomy
Maduako KT, 2022 [30]	25	None	8 weeks	Acute abdominal pain, vomiting	Tubal	Ruptured	Cesarean section at 37 + 5 weeks	Salpingectomy
Kunarathnam V, 2026 [31]	26	None	10 + 6 weeks	Acute abdominal pain	Tubal	Non-ruptured	Term delivery	Laparoscopic salpingectomy
Koutras, A, 2021 [32]	30	None	7 + 4 weeks	Acute abdominal pain, vaginal bleeding	Cervical	Non-ruptured	Uterine curettage	Uterine curettage followed by Foley balloon catheter in cervical canal and vaginal ligation of cervical arteries
Principi G, 2026 [33]	31	None	6 + 5 weeks	Vaginal bleeding	Cervical	Non-ruptured	Cesarean section at 38 + 6 weeks	US-guided cervical curettage
Herrera Castañeda E, 2025 [34]	35	None	9 weeks	Generalized abdominal pain	Cornual	Ruptured	Cesarean section at 39 weeks	Cornual resection
Galatis D, 2025 [35]	29	None	8 weeks	Abdominal pain, vaginal bleeding	Tubal	Ruptured	NA	Salpingectomy

IUP: intrauterine pregnancy; NA: not available; PID: pelvic inflammatory disease; US: ultrasound; GTD: gestational trophoblastic disease; GA: gestational age.

Although most spontaneous HP are diagnosed during the first trimester, exceptional late-presenting cases have also been reported. Tal et al reported that 70% of all HP cases are diagnosed between 5 and 8 weeks of gestation, 20% between 9 and 10 weeks, and only 10% after the 11th week [36]. Momtahan et al described a spontaneous HP consisting of a concurrent intrauterine and abdominal gestation that remained undiagnosed until cesarean delivery at 36 + 4 weeks of gestation [12]. The condition had been misinterpreted as a dichorionic–diamniotic twin pregnancy throughout antenatal follow-up, and the diagnosis was established only intra-operatively. Remarkably, both fetuses were delivered alive, highlighting the wide spectrum of clinical presentation and the potential for delayed diagnosis even in advanced pregnancy.

Abdominal or pelvic pain was the most common reported presenting symptom among the reviewed cases and was frequently accompanied by vaginal bleeding. In several patients, the diagnosis was established only during surgical exploration. Common misdiagnoses included acute appendicitis, ruptured ovarian cyst, and other adnexal masses [7, 18, 31]. In cases complicated by EP rupture and hemoperitoneum, patients often presented with signs of hemorrhagic shock. Nevertheless, atypical manifestations have also been reported, including chest pain, dyspnea, and syncope, further contributing to the diagnostic challenge [22].

Because abdominal pain, vaginal bleeding, adnexal tenderness, and hemodynamic instability may occur in a variety of clinical settings, HP should be included in the differential diagnosis of all symptomatic early pregnancies, particularly when symptoms appear disproportionate to an otherwise apparently reassuring IUP. The differential diagnosis of HP is broad and includes several obstetric and gynecological conditions with overlapping clinical presentations. The key distinguishing features are summarized in Table 3.

**Table 3:** Differential Diagnosis of Heterotopic Pregnancy and Distinguishing Features

Condition	Overlapping features with HP	Distinguishing clues
Threatened miscarriage	Pelvic pain, vaginal bleeding	Adnexal mass, free fluid, persistent pain disproportionate to IUP findings
Hemorrhagic corpus luteum cyst	Adnexal pain, complex adnexal mass	Imaging evolution, ectopic sac/yolk sac, hemoperitoneum pattern
Isolated ectopic pregnancy	Pelvic pain, bleeding	Presence of concurrent IUP in HP
Ruptured ovarian cyst	Acute pain, intraperitoneal fluid	Absence of ectopic gestational structures
Ovarian torsion	Acute abdominal pain, adnexal tenderness	Doppler findings, enlarged ovary
Appendicitis	Lower abdominal pain	Gastrointestinal symptoms, inflammatory markers

HP: heterotopic pregnancy; IUP: intrauterine pregnancy.

Tubal implantation was the predominant ectopic location, while ovarian, abdominal, cervical, cesarean scar, and cornual heterotopic pregnancies were reported less frequently. Despite advances in TVUS, a substantial proportion of cases were diagnosed only after rupture of the ectopic component, emphasizing the ongoing difficulty of early recognition and timely intervention.

Similar to many previously reported spontaneous cases, our patient presented with a ruptured tubal pregnancy and hemoperitoneum requiring emergency surgical intervention. Delayed diagnosis remains a major contributor to maternal morbidity, and rupture may occur even at an early gestational age. Therefore, prompt recognition and surgical management are essential to prevent severe hemorrhage and hemodynamic deterioration.

Surgical management remains the cornerstone of treatment in most cases of HP. The therapeutic approach depends on the patient’s hemodynamic status, the location of the ectopic gestation, and the viability of the IUP. In the reviewed cases, salpingectomy was the most frequently performed procedure, most commonly via laparoscopy. However, laparotomy remains an important option in patients presenting with hemodynamic instability, extensive hemoperitoneum, or when rapid surgical control is required. In the present case, emergency laparotomy was considered the most appropriate approach because of the ruptured tubal pregnancy and significant intra-abdominal bleeding.

The outcome of the IUP in heterotopic gestations is highly variable. Several published reports have demonstrated successful continuation of the IUP following treatment of the ectopic gestation, resulting in delivery of healthy newborns at term or near term [9, 11–13, 18–20, 22, 23, 26, 27, 29–31, 33, 34]. Nevertheless, unusual presentations associated with markedly different reproductive outcomes have also been described. Dubbewar et al reported a rare case of HP consisting of a complete hydatidiform mole co-existing with a ruptured EP, necessitating emergency management of both gestations [24]. Similarly, Kassi et al described a spontaneous heterotopic triplet pregnancy, composed of a monochorionic–diamniotic twin intrauterine gestation and a ruptured left EP. Following salpingectomy, the IUP progressed until 32 weeks of gestation, when two live infants were delivered by a cesarean section [37].

In contrast, our patient presented with a non-viable IUP at the time of diagnosis, in addition to a ruptured tubal gestation. Consequently, uterine evacuation was performed simultaneously with surgical treatment of the EP. These observations underscore the considerable heterogeneity of reproductive outcomes in HP and highlight the importance of individualized management based on maternal condition, gestational viability, and the location of the ectopic gestation.

The present case adds to the limited literature on spontaneous HP occurring in the absence of identifiable risk factors. It further emphasizes that confirmation of an IUP should not preclude meticulous assessment of the adnexa. Early diagnosis remains crucial for preventing maternal morbidity and life-threatening complications, particularly in patients presenting with abdominal pain, vaginal bleeding, or free intraperitoneal fluid on ultrasound examination.

### Conclusions

HP remains an uncommon but potentially life-threatening obstetric condition and should be recognized as a time-sensitive gynecological emergency, particularly when associated with tubal rupture and hemoperitoneum. Although its incidence has increased in parallel with the expansion of ART, spontaneous HP remains exceptionally rare and continues to represent a major diagnostic challenge due to its non-specific presentation and the false reassurance frequently generated by the presence of an IUP. This case underscores the importance of maintaining a high index of suspicion and performing systematic adnexal assessment in all symptomatic early pregnancies, as timely diagnosis and prompt surgical management are essential to reduce maternal morbidity and optimize pregnancy outcomes whenever the IUP is viable.

### Learning points

HP can occur after spontaneous conception and in the absence of traditional risk factors, making clinical suspicion essential even in apparently low-risk patients. The presence of an IUP should never exclude systematic assessment of the adnexa, as concurrent EP may be missed during early ultrasonography. In symptomatic first-trimester patients, particularly those with abdominal pain, vaginal bleeding, free intraperitoneal fluid, or hemodynamic instability, HP should remain part of the differential diagnosis. Early recognition and prompt surgical management are critical to prevent tubal rupture, hemoperitoneum, and severe maternal morbidity.

## Data Availability

Data presented in this study are available on request from the corresponding author due to privacy concerns.

## Abbreviations

HP: heterotopic pregnancy
EP: ectopic pregnancy
IUP: intrauterine pregnancy
ARTs: assisted reproductive technologies
IVF: *in vitro* fertilization
TVUS: transvaginal ultrasound
MRI: magnetic resonance imaging
PID: pelvic inflammatory disease
FGM: female genital mutilation
NA: not available
CRL: crown-rump length
G4P3: gravida 4 para 3
